# Self-Assembled Lecithin-Chitosan Nanoparticles Improved Rotigotine Nose-to-Brain Delivery and Brain Targeting Efficiency

**DOI:** 10.3390/pharmaceutics15030851

**Published:** 2023-03-05

**Authors:** Paramita Saha, Prabhjeet Singh, Himanshu Kathuria, Deepak Chitkara, Murali Monohar Pandey

**Affiliations:** 1 Industrial Research Laboratory, Department of Pharmacy, Birla Institute of Technology & Science, Pilani Rajasthan 333031, India; 2 Nusmetics Pte Ltd., E-Centre@Redhill, 3791 Jalan Bukit Merah, Singapore 159471, Singapore

**Keywords:** rotigotine, lecithin-chitosan hybrid nanoparticles, intranasal delivery, nose-to-brain uptake, brain distribution study

## Abstract

Rotigotine (RTG) is a non-ergoline dopamine agonist and an approved drug for treating Parkinson’s disease. However, its clinical use is limited due to various problems, viz. poor oral bioavailability (<1%), low aqueous solubility, and extensive first-pass metabolism. In this study, rotigotine-loaded lecithin-chitosan nanoparticles (RTG-LCNP) were formulated to enhance its nose-to-brain delivery. RTG-LCNP was prepared by self-assembly of chitosan and lecithin due to ionic interactions. The optimized RTG-LCNP had an average diameter of 108 nm with 14.43 ± 2.77% drug loading. RTG-LCNP exhibited spherical morphology and good storage stability. Intranasal RTG-LCNP improved the brain availability of RTG by 7.86 fold with a 3.84-fold increase in the peak brain drug concentration (C_max(brain)_) compared to intranasal drug suspensions. Further, the intranasal RTG-LCNP significantly reduced the peak plasma drug concentration (C_max(plasma)_) compared to intranasal RTG suspensions. The direct drug transport percentage (DTP (%)) of optimized RTG-LCNP was found to be 97.3%, which shows effective direct nose-to-brain drug uptake and good targeting efficiency. In conclusion, RTG-LCNP enhanced drug brain availability, showing the potential for clinical application.

## 1. Introduction

Parkinson’s disease (PD) is the second most prevalent neurodegenerative disorder, following Alzheimer’s disease [1]. It significantly impairs patients’ quality of life and productivity. Although PD is primarily a motor condition, research indicates that most patients (>90%) also experience non-motor symptoms. Frequently used drugs to treat PD provide only symptomatic relief. The efficacy of most anti-Parkinson drugs is limited due to their low systemic bioavailability and poor availability into the brain.

Rotigotine (RTG) is a dopamine agonist approved for treating PD [2]. It impacts both the motor and non-motor symptoms of PD. The benefits of RTG are superior to other standard dopamine agonists. RTG is available as 1–8 mg transdermal sustained-release patches applied for 24 h. RTG has low (<1%) oral bioavailability due to high hepatic first-pass metabolism [3]. The transdermal patch shows a systemic bioavailability of approximately 37% [4]. The bioavailability also varies depending on the site of application of the transdermal patch [3]. The availability of RTG in the brain is further hampered due to the blood–brain barrier (BBB). Therefore, developing a suitable delivery system for RTG that can be administered via an alternate route is required to avoid first-pass metabolism and efficiently increase the brain availability of the drug.

The BBB hinders the brain’s availability of drugs, affecting their efficacy. Several delivery systems have been developed to overcome the BBB, such as cerebral implants and intracerebroventricular injections, which increase the brain availability of drugs. However, the invasive nature of these methods comes with a considerable risk [5,6]. Intranasal (i.n.) delivery is a non-invasive approach that avoids the BBB by increasing the direct nose-to-brain uptake of drugs. It also offers ease of administration, quick onset of action, and avoids first-pass metabolism and systemic toxicity. It has been reported that olfactory neural pathways and the trigeminal nerves are involved in the transport of drugs to the brain via the nasal cavity [7,8]. However, mucociliary clearance prevents drug retention in the nasal cavity, affecting the direct nose-to-brain delivery of drugs. Therefore, a suitable formulation approach is required to slow the mucociliary clearance process and increase the drug’s permeability via the nasal epithelium following i.n. administration. Several nanocarriers have been investigated for RTG to increase the brain bioavailability of the drug [9,10,11,12]. Considering the advantages and disadvantages of previously reported polymeric and lipidic systems, a self-assembling system containing both polymer and lipid was found to be advantageous as compared to the previous approaches. Chitosan (CS)-lecithin nanoparticles of hydrophilic and lipophilic drugs are reported in the literature for oral, transdermal, and intranasal delivery [13,14,15,16,17]. These nanoparticles have been found to enhance the drugs’ oral, systemic, and brain bioavailability.

CS is a biodegradable polysaccharide with a positive charge. It is a widely used pharmaceutical excipient because it is biocompatible and mucoadhesive. CS is available in various molecular weights, degrees of deacetylation and viscosities. CS-based i.n. formulations have shown improved nasal residence time and better mucoadhesion [18]. CS-nanocarriers are known to affect the tight junctions that help to enhance the transport of nanocarriers via an olfactory neuronal pathway [19]. CS-based nanocarriers with a size of ~100 nm renders increased rate and extent of drug uptake following i.n. administration via either paracellular or transcellular transport [20]. Several other reports also show that the surface and size properties of nanoparticles play a key role in the transport and uptake via the intranasal route, affecting drug brain availability [9,21]. Soy lecithin is a negatively charged phospholipid combination consisting mainly of phosphatidylcholines. It is a biocompatible, safe, and non-immunogenic excipient. The interaction between lecithin and the CS can produce nanoparticles by self-assembling utilizing ionic interactions.

In this study, RTG-loaded lecithin-CS nanoparticles (RTG-LCNP) were prepared and optimized for i.n. administration. This study hypothesized that RTG-LCNP could improve nasal mucoadhesion due to CS and improve RTG brain absorption. RTG-LCNP were characterized for size distribution, the zeta potential, microscopic morphology, drug crystallinity, and in vitro drug release. An ex vivo nasal study was performed to evaluate the RTG nasal permeability from RTG-LCNP. The nasal clearance time and in vivo study of the optimized LCNP were performed to assess the direct nose-to-brain delivery and brain targeting efficiency.

## 2. Materials and Methods

### 2.1. Materials

Mylan Laboratories (Hyderabad, India) provided RTG as a gift sample. Glipizide, an internal standard (IS), was acquired from TCI Chemicals Pvt Ltd. (Chennai, India). Isoflurane USP was purchased from Abbott (Mumbai, India) for inhalation anesthesia. Medium-molecular-weight chitosan (75–85% deacetylated), acetic acid glacial was purchased from SISCO Research Laboratories (SRL) Pvt. Ltd. (Delhi, India). Lecithin (Lipoid S 100, soybean lecithin with phosphatidylcholine) and Poloxamer 407 were obtained as gift samples from Lipoid (GmBH, Germany) and BASF (Mumbai, India), respectively. Sodium chloride, potassium chloride, mannitol, and different buffer salts (KH_2_PO_4_, K_2_HPO_4_) were acquired from SD Fine Chemicals Pvt. Ltd. (Mumbai, India). HPLC grade acetonitrile (ACN) was purchased from Merck (Mumbai, India). Milli-Q water was taken from a Milli-Q^®^ Reference water purification system (GmbH, Germany) and was used in all experimental procedures and analysis. Wistar rats were acquired from Central animal facility, BITS Pilani, Pilani, India. All the statistical analysis were performed using GraphPad Prism 7.0 (GraphPad software Inc., San Diego, CA, USA).

### 2.2. Preparation of Rotigotine Nanoparticles

**Figure 1:**
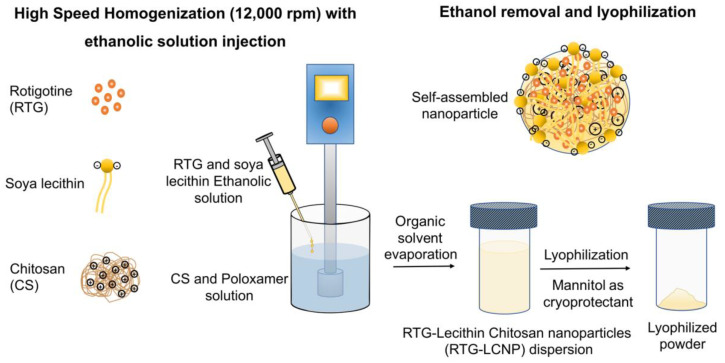
Schematic of RTG-LCNP preparation with soya lecithin, chitosan, and Poloxamer using high-speed homogenization. The soya lecithin and chitosan self-assembled due to ionic interaction upon ethanolic injection with homogenization, encapsulating RTG, and stabilized by Poloxamer.

The LCNP was prepared by a solvent injection method [16,17]. An ethanolic solution of the drug and lecithin was prepared by dissolving 20 mg of RTG and lecithin in 1 mL of ethanol. CS and Poloxamer 407 were dissolved in aqueous acidic solution prepared with glacial acetic acid. The ethanolic solution was injected into the aqueous phase using a 22G needle attached to a polypropylene syringe. The injection was performed for 5 min under high-speed homogenization (Polytron PT 1300D, Kinematica, Lucerne, Switzerland) at 12,000 rpm. The organic solvent was evaporated from nano-dispersion using rotavapor (Buchi, Mumbai, India) for 10 min. After removal of organic solvent, RTG-LCNP was ultracentrifuged (Thermo Fisher, Waltham, MA, USA) at 45,000 rpm for 1 h at 4 °C to attain pellet of RTG-LCNP. The supernatant was decanted, and the LCNP was collected. Further, LCNP pellets were washed thrice with Milli-Q water to remove any traces of free drug from the surface of LCNP. For lyophilization, the finally collected pellet of RTG-LCNP was re-dispersed in mannitol solution (10% *w*/*v*), where mannitol acted as a cryoprotectant (Figure 1). The lyophilized RTG-LCNP was stored under refrigerated conditions (2–8 °C) till further use. A control RTG suspension was prepared by dispersing RTG in 0.2% *w*/*v* methyl cellulose (400 cps).

The effect of several formulation parameters, viz. the ratio of drug:lecithin, the ratio of lecithin:CS, amount of Poloxamer 407, pH of CS solution on particle size, PDI, % entrapment efficiency (%EE), and % drug loading (%DL) were optimized to select the final LCNP batch. The final optimum composition of RTG-LCNP was 20 mg RTG, 60 mg lecithin, 2 mg chitosan, and 5 mg Poloxamer.

### 2.3. Size and Zeta Potential Measurements

The average particle size (d.nm) and PDI of RTG-LCNP were determined using the dynamic light scattering technique (Zetasizer nano ZS, Malvern Instruments, Malvern, UK). The zeta potential was measured using electrophoretic dynamic light scattering. The LCNP suspensions were diluted 10 fold with sodium acetate buffer (pH 5.5) and allowed to equilibrate for 2 min at 25 °C before each measurement. Three measurements were performed for every LCNP suspension, and the mean values were reported for the final particle size, PDI, and zeta potential.

### 2.4. Entrapment Efficiency and Drug Loading 

The entrapment efficiency (%EE) was estimated from the unentrapped amount of RTG (*W_Free drug_*). The free RTG was separated from LCNP suspension by ultracentrifuged at 45,000 rpm for 1 h. The supernatant was analyzed using the validated RP-HPLC analytical method to measure free RTG (*W_free drug_*) [22]. %EE of LCNP was calculated using the following equation:

(1) $$\%EE=\left(\frac{W_{Total\;drug}-W_{Free\;drug}}{W_{Total\;drug}}\right)\times 100$$

where *W_Total drug_* is the total amount of RTG used in the preparation of RTG-LCNP, *W_Free drug_* is the unentrapped drug.

The drug loading (%DL) was estimated following the direct method. RTG-LCNP pellets were collected after ultracentrifugation (45,000 rpm for 1 h at 4 °C), washed, and dried under vacuum. The collected pellets were first weighed, dissolved in ACN to extract RTG, and diluted with the mobile phase. The quantity of RTG was determined using validated RP-HPLC analytical method [22]. Finally, %DL was calculated using the formula given in the equation below:

(2) $$\%DL=\left(\frac{W_{RTG}}{W_{RTG-LCNP}}\right)\times 100$$

where *W_RTG_* is the weight of RTG loaded in the LCNP and *W_RTG-LCNP_* is the total weight of NP.

### 2.5. Differential Scanning Calorimetry

Thermal analysis was carried out using differential scanning calorimetry (DSC) to analyze the physical state of RTG encapsulated in the optimized RTG-LCNP. Lyophilized RTG-LCNP was accurately weighed inside an aluminum pan and crimped. The samples were analyzed using DSC-60 Plus (Shimadzu, Nakagyo-ku, Kyoto, Japan) at a temperature range of 30–250 °C and heated at a rate of 5 °C/min in a nitrogen environment (50 mL/min). DSC analysis was also performed for pure RTG, CS, lecithin, and mannitol.

### 2.6. Field Emission Scanning Electron Microscopy

A field emission scanning electron microscope (FESEM) (FEI, Hillsboro, OR, USA) was used for the examination of surface morphology of the optimized LCNP. Briefly, 5 μL of optimized RTG-LCNP suspension was dropped onto a glass coverslip and left overnight to dry under the desiccator [23]. The sample containing glass coverslip was attached to the aluminum stab using double-sided carbon tape. Finally, the samples were sputter coated for 50 s by Q150TES sputter coater (Quorum Technologies, Laughton, East Sussex, UK). Gold-coated samples were analyzed using FESEM using a 15 kV high-voltage vacuum pump.

### 2.7. Transmission Electron Microscopy

The particle size and shape of RTG-LCNP and pure-RTG were evaluated using transmission electron microscopy (TEM) (JEOL Ltd., Akishima, Tokyo, Japan) at an accelerating voltage of 120 kV. The samples were prepared by drop cast on carbon grids. The formulation droplet was dropped on the carbon grid and casted on the grid for a few minutes. Then, the excess liquid was soaked using blotting paper before analysis. The cast grid was placed in TEM to take microscopic images for morphological analysis.

### 2.8. Storage Stability of Nanoparticles

The storage stability of the lyophilized RTG-LCNP was analyzed over 60 days in refrigerated conditions. RTG-LCNP were taken in airtight glass containers (15 mL) and stored at 2–8 °C. Samples (n = 3) were collected on 7 day, 30 day, and 60 day, redispersed in Milli-Q water by gentle manual shaking, and evaluated for the particle size (d.nm), PDI, zeta potential (mV), and %DL.

### 2.9. In Vitro Drug Release

RTG-LCNP drug release was performed using the dialysis bag method (Molecular weight cut-off of 12 kDa, Himedia, Mumbai, India). The dialysis bag was soaked in Milli-Q water for 2 h. An amount of 1 mg drug equivalent RTG-LCNP and RTG suspension were separately taken in a dialysis bag. Bags were sealed and immersed in the 50 mL phosphate buffer saline (PBS, pH 7.4). The system was maintained at 37 ± 2 °C with constant stirring at 100 rpm [24]. The 1 mL samples were withdrawn at predetermined intervals from 0.5 h to 24 h, and replenished with pre-heated fresh medium. The samples were diluted with mobile phase and analyzed using the validated RP-HPLC method [22]. The release profiles of RTG-LCNP were analyzed to understand the kinetics and release mechanism. The most common mathematical models, i.e., first-order, Higuchi, and Korsmeyer–Peppas were applied. The high correlation coefficient (R^2^) was taken as the best fit. The ‘n’ value obtained in the Korsmeyer–Peppas model was used to assess the drug release mechanism. The similarity factor (*f_2_*) was determined to compare RTG-LCNP and RTG suspension release profiles.

### 2.10. Ex Vivo Nasal Drug Permeation

Goat nasal mucosa was acquired from a local slaughterhouse. The Franz diffusion cell (Orchid Scientific, Nasik, India) with a diffusion area of 0.785 cm^2^ was used. The mucosa was first hydrated in PBS (pH 6.4) for 15 min 5 mL of PBS (pH 6.4) as permeation media was filled in the receptor compartment. The nasal mucosa was facing toward the donor compartment. The diffusion cell was kept under magnetic stirring at 50 rpm and maintained at 33 ± 1 °C 1 mL of each formulation was placed in the donor compartment to study drug nasal permeation. The 500 μL samples were taken at various intervals, from 5 min to 360 min, and replenished with the pre-heated fresh media. All the samples were centrifuged (Eppendorf^®^, Hamburg, Germany) at 15,000 rpm for 15 min at 4 °C. Supernatants were collected, processed, and analyzed using validated RP-HPLC [22].

### 2.11. In Vivo studies in Wistar Rats

Male Wistar rats aged 9–10 weeks weighing 250–260 g were used. A 2 mg/Kg dose was administered for optimized RTG-LCNP and RTG suspension. Prior approval from the Institute’s Animal Ethics Committee (IAEC) was obtained for all the in vivo animal studies (Protocol number- IAEC/RES/26/07/REV-1/30/19).

#### 2.11.1. Administration of Intranasal (i.n.) Formulation to Rats

Formulations were administered to the nasal cavity of rats using a 1.3 cm long soft cannula (Instech Laboratories, Plymouth, PA, USA) attached in front of microtip. Rats were anaesthetized inside an anesthetic chamber using isoflurane prior to dosing and during plasma collection. A volume of 75 μL of each formulation (dose of 2 mg/Kg) was administered in one of each nostrils [16], and animals were kept in supine position till recovery. The pharmacokinetics (PK) parameters of the brain and plasma of LCNP were compared with those of RTG suspension.

#### 2.11.2. Mucociliary Transport Time RTG-LCNP

After i.n. administration of formulations, the oropharyngeal cavity was swabbed using cotton buds at 5–90 min intervals. For 1 h after study initiation, animals were not fed the food. The samples were diluted 10 fold with the mobile phase and analyzed using a validated analytical method [22]. The time point when the drug was first detected in the oropharyngeal cavity was called mucociliary transport time.

#### 2.11.3. Brain and Plasma PK Analysis

For the brain PK study, rats were divided into various groups with n = 4 in each group. The groups were divided based on time points of brain collection. The rats were sacrificed by cervical dislocation, and the whole brain was collected at predetermined intervals (0.5, 1, 2, 4, 6, and 8 h). At each time point, rats were sacrificed for brain PK studies. A separate group of rats (n = 4) was assigned for the plasma PK study. Blood samples were collected through retro-orbital plexus puncture at predetermined intervals (0, 0.08, 0.25, 0.5, 1, 2, 4, 6, and 8 h). Brain and plasma samples were processed and analyzed using a validated RP-HPLC bioanalytical method (Supplementary Materials S1). PK parameters (viz. C_max_, T_max_, AUC_0→tlast_, MRT, clearance) were determined by non-compartmental analysis (NCA) using Phoenix WinNonlin (Version 8.0) for both brain and plasma.

Drug targeting efficiency percentage (DTE (%)) and brain drug direct transport percentage (DTP (%)) were calculated to evaluate the brain targeting efficiency. DTE (%) signifies the total drug transported to the brain that contains direct nose-to-brain and indirect nose-to-brain via systemic circulation. DTP (%) demonstrates the drug fraction delivered directly to the brain through the nose. DTE (%) > 100 and a DTP (%) > 0, signify substantial direct nose-to-brain distribution of the drug. DTE (%) and DTP (%) were calculated using the following equations:

(3) $$DTE\;\left(\%\right)=\frac{{\left(AUC_{brain}/AUC_{plasma}\right)}_{i.n.}}{{\left(AUC_{brain}/AUC_{plasma}\right)}_{i.v.}}\times 100$$

where AUC_brain_ = AUC_0→tlast_ in brain, AUC_plasma_ = AUC_0→tlast_ in plasma

(4) $$DTP\left(\%\right)=\frac{AUC_{brain}{}_{i.n.}-B_{x}}{AUC_{brain}{}_{i.n.}}\times 100$$

where

$$B_{x}=\frac{AUC_{brain}{}_{i.v.}}{AUC_{plasma}{}_{i.v.}}\times AUC_{plasma}{}_{i.n.}$$

where AUC_brain_ = AUC_0→tlast_ in brain, AUC_plasma_ = AUC_0→tlast_ in plasma. 𝐵_x_ is the fraction of AUC_0→last(brain)_ from systemic circulation (via an indirect pathway) after i.n. administration of a given formulation.

### 2.12. Histopathology of Brain

The brains were isolated from rats before i.n. administration (as control) and at 8 h after i.n. administration of drug suspension and RTG-LCNP. The isolated brains were washed in PBS (pH 7.4) to remove traces of blood and connective tissues. The cleaned brains were weighed and fixed in 10% *v/v* formalin solution. The brain tissues were embedded in paraffin wax, sectioned, and stained with hematoxylin and eosin. The histopathological slides were examined using an inverted light microscope (Carl Zeiss, Jena, Germany). Three rats from each group were used for this study.

## 3. Results and Discussion

### 3.1. Effect of Drug: Lecithin Ratio on Nanoparticle Size

The amount of lecithin played an essential role in preparation of the nanoparticles. Lecithin concentration also directly affect %EE and %DL of nanoparticles. Firstly, drug:lecithin ratio was optimized for preparation of the formulation. The drug:lecithin ratio affected the particle size and PDI of the drug:lecithin dispersion (Table 1). Drug:lecithin ratio was varied between 1:1 to 1:6 (*w*/*w*) during the formulation optimization. The change in drug:lecithin ratio from 1:1 to 1:3 (*w*/*w*) resulted in significant (*p* < 0.0001, one-way ANOVA-Tukey test) decrease in the particle size. Further change in drug:lecithin ratio from 1:4 to 1:6 (*w/w*) caused an increased particle size and PDI significantly (*p* < 0.0001, one-way ANOVA-Tukey test). The ratio of 1:3 (*w*/*w*) had the lowest particle size and PDI. Hence, the drug:lecithin (1:3) ratio was considered the optimum for RTG-LCNP formulation. Both lower and higher lecithin concentrations resulted in increased particle size and PDI. The result might be attributed to the fact that an increase in lecithin amount results in aggregation of particles, whereas a decrease in lecithin amount fails to suitably stabilize the dispersion.

### 3.2. Effect of Lecithin:CS Ratio on the Particle Size and PDI

Lecithin:CS ratio also affects the particle size and PDI of the LCNP. A proper complexation between lecithin and CS is a prerequisite for preparing self-assembled LCNP and resulting in the desired particle size. Lecithin:CS ratio was varied between 10 to 30, where the lecithin amount was kept constant at 60 mg. The increased lecithin:CS ratio resulted in a lower particle size (Table 2), whereas a lower lecithin:CS ratio resulted in a higher particle size. These results could be attributed to formation of larger aggregates at low lecithin:CS ratio [17]. Hence, the lecithin:CS ratio (30) was selected for further optimization of LCNP which demonstrated the lowest particle size.

### 3.3. Effect of the Amount of Poloxamer 407 on the Particle Size, PDI and %EE

Poloxamer 407 can directly affect RTG-LCNP particle size, PDI, and %EE. The drug exhibited lower solubility in Poloxamer 407 than other stabilizers [9]. Thus, Poloxamer 407 was selected as a stabilizer. The amount of Poloxamer 407 was varied from 2.5 to 10 mg by keeping the previous two parameters constant (drug:lecithin ratio: 1:3 (*w*/*w*) and lecithin:CS ratio 30) (Table 3). Both low Poloxamer 407 amount (LCNP 10) and high Poloxamer amount (LCNP 12) showed a significantly (*p* < 0.0001) higher particle size of LCNP than LCNP 11. This can be attributed to insufficient Poloxamer (at low concentration) to stabilize the formulation. However, the high Poloxamer amount can result in higher steric hinderance, negatively affecting interaction of lecithin, chitosan and drug, rendering a high particle size. In addition, changes in drug solubility with poloxamer concentrations could also affect size parameters, whereas an increase in Poloxamer 407 amount negatively affected the %EE (Table 3). The high Poloxamer 407 increased the drug solubility, negatively impacting the %EE of RTG-LCNP. Hence, 5 mg of Poloxamer 407 was selected for the further optimization of %DL of the nanoparticles.

### 3.4. Effect of pH of CS Solution on the Particle Size and %DL

pH of CS solution is already reported to have a significant effect in the preparation of LCNP by ionic gelation method. CS gets solubilized in water due to the ionization of amine group. The positive charge causes the ionic interaction with the negatively charged lecithin [15]. CS solubility is decreased at pH > 6, because of poor ionization of the amine group [25]. Furthermore, in the case of i.n. delivery, the pH of the formulation is an important factor. Formulation pH different from the physiological pH (range) of the nasal cavity irritates the nasal cavity. Additionally, pH of CS solution might affect the solubility of RTG in the aqueous phase and finally effect the %DL. Hence, the pH of CS solution for optimization %DL was varied between pH 5 to 6. The effect of pH of CS solution on %DL of prepared RTG-LCNP is presented in Table 4.

**Figure 2:**
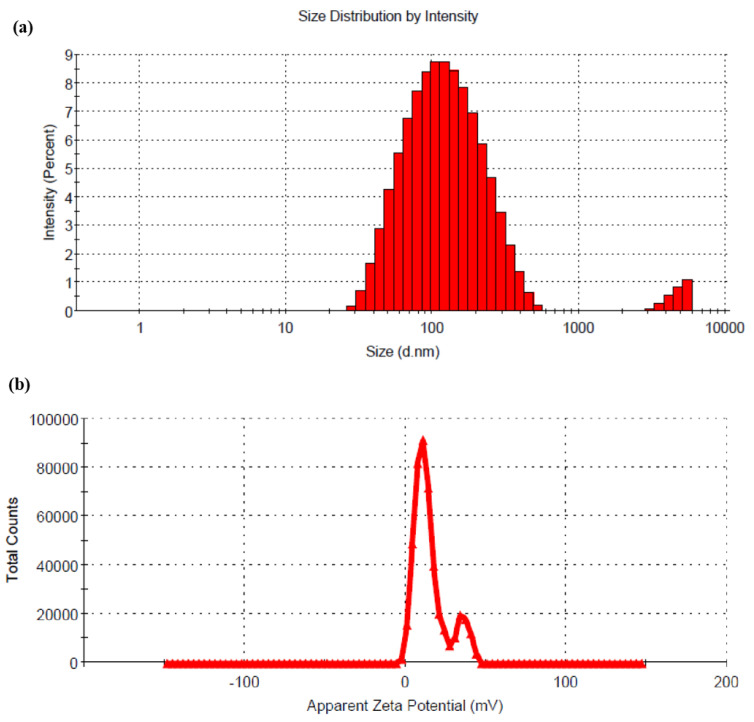
(**a**) Hydrodynamic diameter of optimized RTG-LCNP based on the % intensity; (**b**) zeta potential of optimized RTG-LCNP.

The result showed that with a decrease in pH of CS solution, the %DL was decreased when all the other formulation and process parameters were kept constant. This result might be attributed to the fact that the drug demonstrates a pH-dependent solubility. At lower pH of CS solution, the drug solubility increases, resulting in a lower %DL. RTG is soluble between pH 1 to 5, and with increasing pH, the solubility of the drug decreases. The change in pH (5 to 6) of the CS solution has no significant effect on the particle size of the prepared LCNP. Hence, LCNP 15 which showed a better %DL (14.43 ± 2.77) and a particle size of 108 ± 4 nm (Figure 2a) was selected as the optimal formulation. The %EE for LCNP 15 was 85.22 ± 1.83. The zeta potential of the optimized formulation was 14.9 ± 0.5 mV (Figure 2b).

### 3.5. Differential Scanning Calorimetry

**Figure 3:**
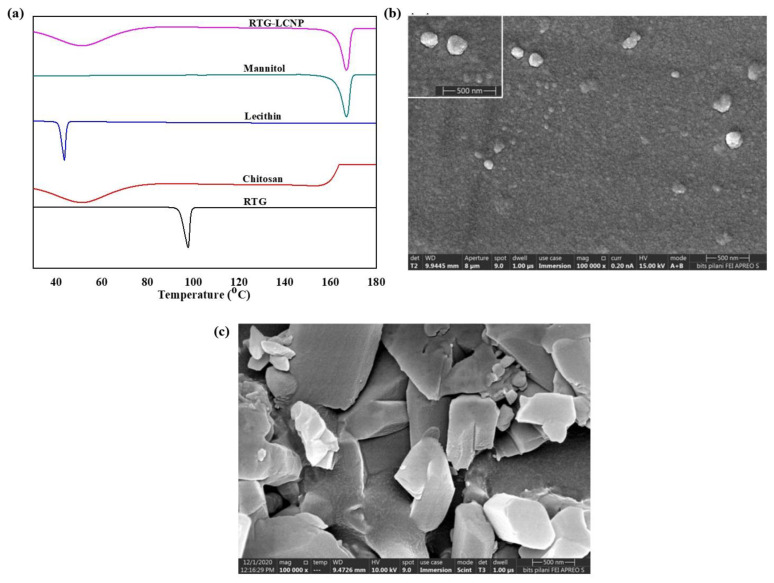
(**a**) DSC thermograms of RTG, Chitosan, Lecithin, Mannitol and lyophilized RTG-LCNP, (**b**) Surface morphology of the optimized RTG-LCNP by FESEM, (**c**) Surface morphology of pure crystalline RTG by FESEM.

DSC thermograms of pure RTG, lecithin, CS, mannitol (cryoprotectant), and lyophilized RTG-LCNP are presented in Figure 3a. The pure RTG showed a sharp endothermic melting peak at 97.87 °C [26], indicating that RTG is crystalline. Thermogram of CS shows no endothermic peak, whereas lecithin exhibits its characteristic sharp endothermic peak at 43.84 °C. Finally, the DSC thermogram of lyophilized RTG-LCNP exhibits a sharp endothermic peak at 166 °C which corresponds to the melting point of the cryoprotectant (mannitol) used for the lyophilization of LCNP [27]. The disappearance of RTG melting peak might be attributed to the entrapment of RTG in RTG-LCNP. The absence of peak might also be due to the conversion of RTG to its amorphous state within the LCNP.

### 3.6. Field Emission Scanning Electron Microscopy

The surface morphology of RTG-LCNP was characterized by FESEM. Figure 3b revealed almost spherical morphology of final RTG-LCNP. FESEM image revealed that the final RTG-LCNP were predominantly uniform in shape with smooth surfaces. The FESEM image also shows spherical particles of RTG-LCNP are of similar size as obtained by dynamic light scattering analysis. Figure 3c shows the FESEM images of crystalline RTG. Figure 3c revealed that the RTG crystal are orthorhombic in shape [28].

### 3.7. Tranmission Electron Microscopy

**Figure 4:**
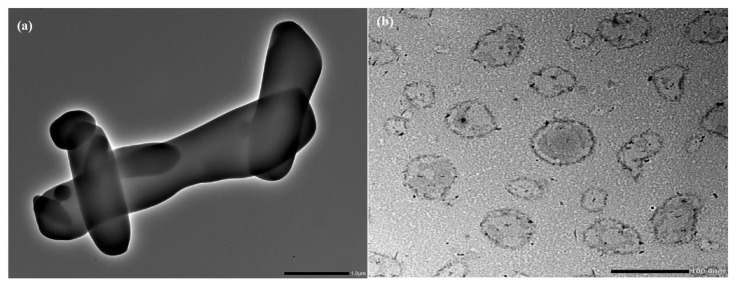
(**a**) Surface morphology of pure RTG by TEM, (**b**) Surface morphology of optimized RTG-LCNP by TEM.

TEM image of pure RTG revealed that the drug was crystalline with a sharp edge Figure 4a. In contrast, the morphology of LCNP showed a nearly spherical shape (Figure 4b). The core of the LCNP was surrounded by a compact outer layer which indicates the formation of a core-shell structure of the optimized LCNP [13,14].

### 3.8. Stability Study of RTG-LCNP

The results obtained from the stability study of RTG-LCNP in refrigerated conditions are shown in Table 5. No significant variation was observed in any of the parameters such as the particle size (d.nm), PDI, zeta potential, and %DL between freshly prepared LCNP and stored LCNP over 60 days (*p* > 0.05).

### 3.9. In Vitro Drug Release

**Figure 5:**
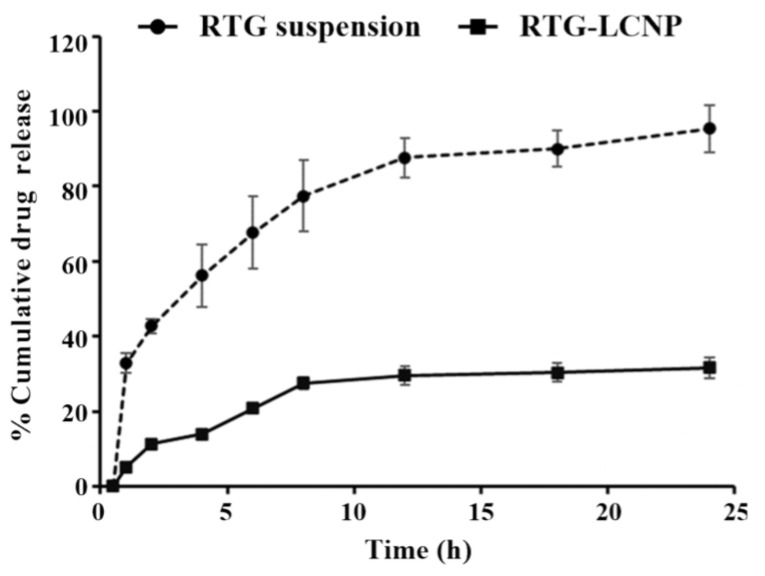
In vitro release profiles of RTG suspension and optimized RTG-LCNP (LCNP 15) in PBS (pH 7.4).

The in vitro release of pure RTG suspension and optimized RTG-LCNP were performed, and the profile is presented in Figure 5. From the drug suspension, almost 100% were released within 24 h. While from optimized LCNP, RTG released in a controlled pattern up to 24 h. Form RTG-LCNP percentage cumulative drug released (%CDR) was up to 32.44 ± 2.71% after 24 h. The drug release from RTG-LCNP showed best fit with the Korsmeyer–Peppas model with R^2^ of 0.9296. The R^2^ with first-order was 0.3526, and R^2^ with the Higuchi model was 0.8289. The drug release mechanism from LCNP was best explained as non-Fickian diffusion type (n = 0.331) [29]. Similar results were also found in several literature reports. Alomrani et al. showed that lipophilic 5-fluorouracil-loaded chitosan-coated flexible liposomes exhibited Korsmeyer–Peppas model-dependent release profile [30]. Ilk et al. and Murthy et al. have reported that lecithin CS delivery system of lipophilic drugs demonstrated Korsmeyer–Peppas model-dependent release profile [17,31]. Additionally, a lower similarity factor (*f_2_* = 20) also demonstrated that the release profiles of RTG-LCNP and RTG are not similar to each other.

### 3.10. Ex Vivo Nasal Permeation

**Figure 6:**
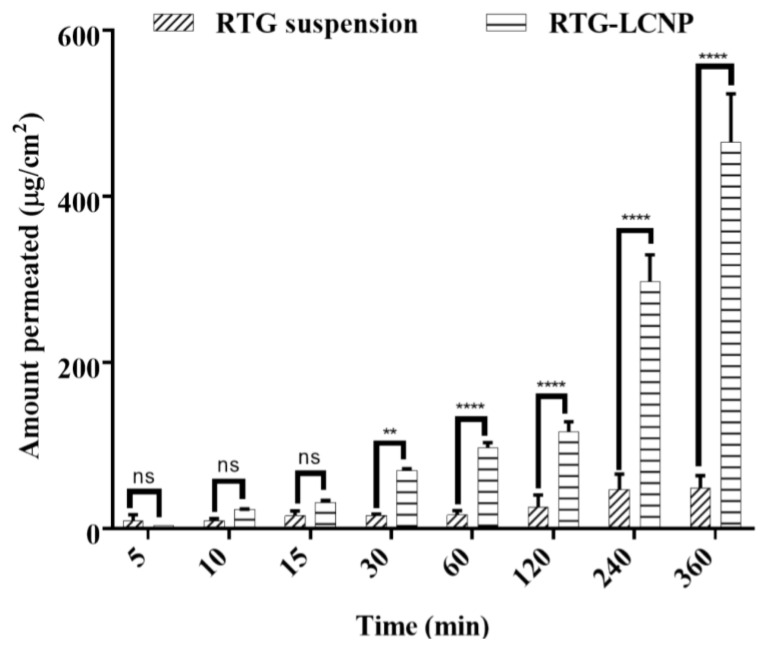
Ex vivo amount of drug permeated/unit area from optimized RTG-LCNP (LCNP 15) and drug suspension via goat nasal mucosa (n = 3, Mean ± SD). ‘ns’ indicates no significant difference, ‘**’ indicates p-value ≤ 0.01 ‘****’ indicates p-value ≤ 0.0001.

An ex vivo nasal permeation study was performed to observe the permeation behaviour of pure RTG suspension and RTG-LCNP. The mean cumulative ex vivo RTG permeated per unit area vs. time through the goat nasal mucosa is presented in Figure 6. The permeation profile revealed that RTG amount permeated (464.89 ± 58.22 μg/cm^2^) from LCNP was significantly higher than that of the pure drug suspension (*p* < 0.05). Optimized RTG-LCNP showed a 9.66-fold increase in the amount permeated compared to the pure drug suspension. The result indicated that the LCNP formulation provides better permeability than pure drug. Better permeation of RTG from LCNP can be attributed to the presence of CS in the formulation. CS in the formulation might improve the ex vivo nasal permeation via paracellular transport by opening the tight junction of the biological membrane [14,32,33].

### 3.11. In Vivo Studies

#### 3.11.1. Mucociliary Transport Time of Nanoparticles

**Figure 7:**
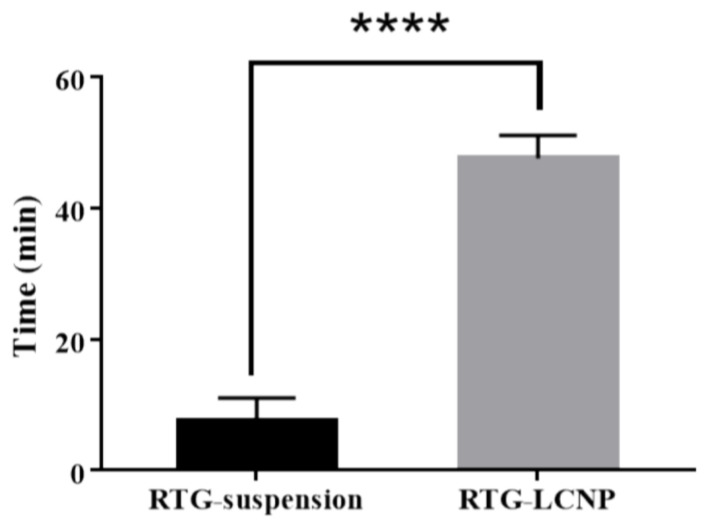
Mucociliary transport time of aqueous RTG suspension and RTG-LCNP (LCNP 15). Student’s independent t-test with one-tail was applied. ‘****’ shows *p*-value <0.05.

Mucociliary transport time for pure RTG suspension and RTG-LCNP was 7.5 ± 3.53 min and 47.5 ± 3.53 min, respectively. RTG-LCNP demonstrated a higher (*p* < 0.05) mucociliary transport time than that RTG suspension (Figure 7). The increased mucociliary transport time of RTG-LCNP compared to the pure drug suspension indicates a higher residence time in the nasal cavity. The result might be attributed to the presence of mucoadhesive CS in the formulation. The high mucociliary transport time of RTG-LCNP indicated that the nanocarrier could resist the mucociliary clearance process and increased the retention time in the nasal cavity.

#### 3.11.2. Plasma and Brain PK Analysis

**Figure 8:**
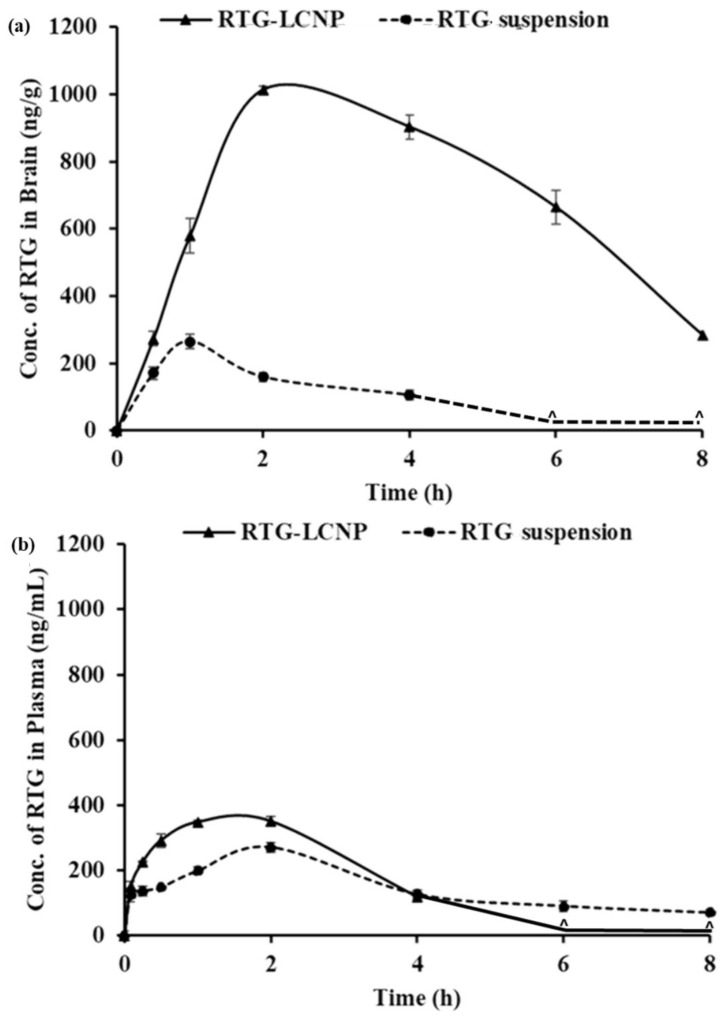
PK profiles of RTG attained after i.n. administration of RTG-LCNP (LCNP 15) and RTG suspension in (**a**): Brain and (**b**): Plasma. ‘^’ in both the profiles denote that the concentration of RTG was not detected at those time points in brain matrices and plasma.

The PK parameters of RTG-LCNP and the pure drug suspension are given in Table 6. Plasma AUC_0→tlast_ and C_max_ for both formulations were not significantly different. The brain AUC_0→tlast_ for RTG-LCNP was approximately 8.77-fold higher than that of the brain AUC_0→tlast_ of the pure drug suspension. The brain C_max_ of RTG-LCNP showed a 3.83-fold increment as compared to pure RTG suspension after i.n. administration. An unpair t-test comparison for brain AUC_0→tlast_ for both formulations showed a statistically significant (*p* < 0.0001) difference between the two formulations. The brain concentrations of RTG were compared between RTG-LCNP and pure RTG suspension using *t*-tests for all the time points. The drug concentrations from RTG-LCNP in the brain were significantly higher than RTG suspension at all respective time points at a 5% confidence interval (Figure 8a). To further evaluate the in vivo performance of LCNP formulation, DTP (%) and DTE (%) were calculated as per Equation 3 and 4. Here, as a systemic route, the i.v. administration was used. The DTE (%) for RTG-LCNP was 3673.7, significantly higher than 100. This result implies that brain exposure of LCNP after i.n. administration is superior to that attained via the systemic route. This result finally indicates the nose-to-brain uptake efficacy of the prepared LCNP. The DTP (%) was 97.3 for RTG-LCNP, showing effective direct nose-to-brain uptake of RTG to the brain. The high and positive DTE (%) and DTP (%) of RTG-LCNP may be ascribed to the better retention of formulation at the site of administration than drug suspension. CS in LCNP formulation improved mucoadhesion in the nasal cavity [34]. The presence of CS might further help in reversibly opening tight junctions, facilitating drug uptake to the brain via the olfactory nerve pathway through paracellular transport [35]. Similar results were observed by few other researchers. Bhattamisra et al. showed a relatively high DTP (%) value (53.87 ± 10.14) when RTG was loaded in CS nanoparticles compared to RTG solution [12]. Md et al. reported that bromocriptine-loaded i.n. CS nanoparticles showed a high DTE (%) value (265.6 ± 37.3) compared to drug solution [36]. Wang et al. reported that RTG-loaded micellar thermosensitive gel improved DTP (%) in olfactory bulb, cerebrum, cerebellum, and striatum within a range of 49–70% [11]. These results confirm that the mucoadhesive properties of nanocarriers or a nanocarrier embedded in situ gel which has the ability to sustain the mucociliary clearance in the nasal cavity and also ability to open the tight junctions result in better brain bioavailability of the drugs via i.n. administration.

Plasma AUC_0→tlast_ from RTG-LCNP was significantly higher than drug suspension (Table 6). This result might be attributed to LCNP via an indirect pathway. RTG also reached the brain in higher amounts. CS present in the formulation might facilitate LCNP reaching the brain from systemic circulation by passing through the BBB [32,33]. The presence of CS in LCNP might also result in opening a tight junction of the nasal epithelium, which finally leads to better systemic exposure of drug from LCNP from the respiratory region compared to pure suspension. The high plasma drug concentration from LCNP might be another reason for high DTE (%).

### 3.12. Histopathology of Brain

**Figure 9:**
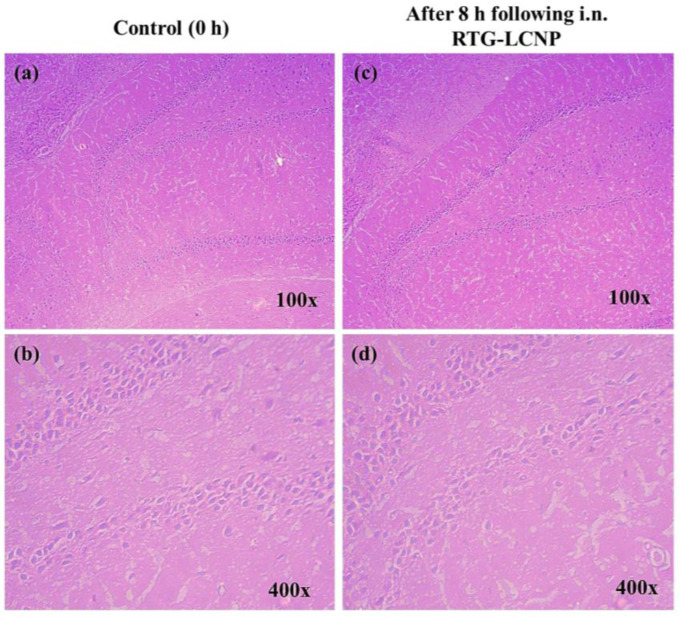
Histopathological evaluations of brain (hippocampal region) in different conditions: (**a**) Control animal at 100× magnification, (**b**) control animal at 400× magnification, (**c**) LCNP treated animal at 100× magnification, and (**d**) LCNP treated animal at 400× magnification.

At 0 h (control) and 8 h after receiving RTG-LCNP (treated), the morphology of the hippocampus region on brain slides was investigated for any toxicity (Figure 9) [37]. The morphology of the hippocampal area of LCNP the treated rat (Figure 9c,d) was similar to that of the control (Figure 9a,b). Figure 9d demonstrated that the in the hippocampal region, there were no indications of neuronal damage, such as cell body necrosis or shrinking. This suggested that RTG-LCNP did not cause any damage to the brain and was safe for clinical use.

## 4. Conclusions

Critical formulation variables of RTG-LCNP were optimized for an anticipated particle size, PDI, %EE, and %DL. The optimized RTG-LCNP was stable in refrigerated conditions for at least 2 months. The LCNP showed higher nasal permeation and mucociliary transport time than RTG suspension. RTG-LCNP improved PK parameters with high brain bioavailability and good targeting efficiency. Overall, RTG-LCNP can improve brain delivery following i.n. administration and has the potential for clinical application.

## Figures and Tables

**Table 1:** Effect of drug:lecithin ratio on the particle size and PDI of RTG-LCNP.

Formulation Code ^a^	Drug:Lecithin Ratio (*w*/*w*)	Particle Size (nm)	PDI
LCNP 1	1:1	220 ± 1.33	0.451 ± 0.011
LCNP 2	1:2	182 ± 2.34	0.412 ± 0.014
LCNP 3	1:3	123 ± 2.12	0.292 ± 0.002
LCNP 4	1:4	263 ± 1.22	0.409 ± 0.009
LCNP 5	1:5	294 ± 1.56	0.495 ± 0.003
LCNP 6	1:6	322 ± 2.86	0.309 ± 0.001

^a^ LCNP 1 to LCNP 6 contain 20 mg of RTG

**Table 2:** Effect of lecithin:CS ratio on the particle size and PDI of RTG-LCNP (n = 3).

Formulation Code ^a^	Lecithin:CS Ratio	Particle Size (nm)	PDI
LCNP 7	10	203.6 ± 1.22	0.430 ± 0.001
LCNP 8	20	171.0 ± 2.31	0.394 ± 0.002
LCNP 9	30	102.0 ± 1.22	0.312 ± 0.006

^a^ LCNP 7 to LCNP 9 contain 20 mg of RTG.

**Table 3:** Effect of the amount of Poloxamer 407 on the particle size, PDI, and %EE of RTG-LCNP (n = 3). One-way ANOVA with Tukey test was applied. *p*-value ≤ 0.05 was considered significant.

Formulation Code ^a^	Amount of Poloxamer 407 (mg)	Particle Size (nm)	PDI	%EE
LCNP 10	2.5	259.8 ± 5.17	0.309 ± 0.009	93.1 ± 3.61
LCNP 11	5	110.3 ± 1.09 ****	0.348 ± 0.012	87.6 ± 2.93
LCNP 12	10	193.7 ± 4.05 ****	0.421 ± 0.018	83.2 ± 1.90

^a^ LCNP 10 to LCNP 12 contain 20 mg of RTG. ‘*’ indicates levels of significance in comparison to LCNP 10. ‘****’ indicates *p*-value ≤ 0.0001.

**Table 4:** Effect of pH of CS solution on the particle size and %DL of RTG-LCNP.

Formulation Code ^a^	pH	Particle Size (nm)	%DL
LCNP 13	5.0	102.0 ± 0.0	6.33 ± 3.35
LCNP 14	5.5	107.8 ± 2.0	10.72 ± 4.03
LCNP 15	6.0	108.0 ± 4.0	14.43 ± 2.77

^a^ LCNP 13 to LCNP 15 contain 20 mg of RTG, a lecithin CS ratio of 30 and Poloxamer 407 of 5 mg.

**Table 5:** Stability data of lyophilized RTG-LCNP powder.

Parameters	0 Day	7 Day	30 Day	60 Day
Particle size (d.nm)	108.2 ± 4.40	105.1 ± 4.38	103.3 ± 1.56	119.8 ± 11.10
PDI	0.312 ± 0.001	0.310 ± 0.002	0.297 ± 0.022	0.371 ± 0.325
Zeta potential (mV)	14.9 ± 0.5	14.1 ± 0.3	13.8 ± 0.3	16.2 ± 0.3
%DL	14.43 ± 2.77	14.75 ± 0.12	15.01 ± 2.39	12.85 ± 4.03

**Table 6:** Plasma and brain PK parameters for RTG-LCNP (LCNP 15) and RTG suspension after i.n. administration.

PK Parameters	Brain		Plasma	
	RTG-LCNP	RTG Suspension	RTG-LCNP	RTG Suspension
AUC_0→tlast_ (ng*h/g) ^b^, (ng*h/mL) ^p^	5507.57± 23.91	628.11 ± 12.21	1060.44 ± 29.95	779.01 ± 14.11
C_max_ (ng/g) ^b^, (ng/mL)^p^	1013.47 ± 11.28	264.71 ± 21.12	230.87 ± 8.19	270.12 ± 18.50
T_max_ (h)	2 ± 0.03	1 ± 0.01	1 ± 0.02	2 ± 0.01
MRT (h)	3.81 ± 0.38	1.82 ± 0.15	1.58 ± 0.05	3.15 ± 0.81
Clearance (g/h) ^b^, (mL/h) ^p^	78.57 ± 12.19	-	-	312.65 ± 15.59

^b^ unit for brain PK parameters; ^p^ unit for plasma PK parameters. RTG dose for both i.n. formulations: 2 mg/Kg; for plasma PK n = 4 animals were used, and n = 4 animal brains were used for brain PK at every time point. The brain and plasma data are represented as the mean ± SD.

## Data Availability

Data can be provided on request.

## Supplementary Materials

The following supporting information can be downloaded at: https://www.mdpi.com/article/10.3390/pharmaceutics15030851/s1, S1: Processing of brain and plasma matrices.
