# Supplementary material for: Self-Assembled Lecithin-Chitosan Nanoparticles Improved Rotigotine Nose-to-Brain Delivery and Brain Targeting Efficiency
Source: Pharmaceutics. 2023 Mar 5;15(3):851. doi: 10.3390/pharmaceutics15030851 (PMC10052746; doi:10.3390/pharmaceutics15030851)
Supplement: Supplementary file 1 [file pharmaceutics-15-00851-s001.zip › pharmaceutics-2123653-supplementary.pdf]

pharmaceutics

Supplementary Materials: Self-Assembled Lecithin-Chitosan Nanoparticles Improved Rotigotine Nose-to-Brain Delivery and Brain Targeting Efficiency

Paramita Saha, Prabhjeet Singh, Himanshu Kathuria, Deepak Chitkara and Murali Monohar Pandey

S1: Processing of brain and plasma matrices

To separate plasma, blood was collected in centrifuge tubes (Tarson, Kolkata, India) containing 4.5% (w/v) disodium EDTA as an anticoagulant and centrifuged (Eppendorf®, Hamburg, Germany) at 7500 rpm for 10 min at 4 °C. For brain homogenate, brain samples were weighed, PBS (pH 7.4) was added in 1:3 ratios, and the mixture was homogenized for 5 minutes at 12,000 rpm (Kinematica, GmBH, Germany). To separate brain matrices, the brain homogenate was centrifuged (Eppendorf®, Hamburg, Germany) at 7500 rpm for 10 minutes at 4 °C. Separately, 90 mL of brain matrices and 90 μL of plasma were combined with 10 μL of an internal standard (Glipizide) solution. Protein was precipitated using acetonitrile (ACN). ACN was added, and 300 μL was vortexed for 7 minutes. To extract protein-free brain matrices and plasma, the samples were centrifuged (Eppendorf®, Hamburg, Germany) at 15,000 rpm for 10 min at 4 °C. Supernatants were placed in microcentrifuge tubes (Tarsons, Kolkata, India) and evaporated under vacuum. The dried samples were reconstituted with 100 μL of mobile phase and vortexed (Tarsons, Kolkata, India) for 5 min. Finally, samples were put into HPLC vials and injected onto the column for quantification using a validated RP-HPLC.

A RP-HPLC system with a pulse-free solvent system with two pumps, a 5-line degasser, a block heating type column oven, a sample chiller, and an intelligent auto-sampler with a UV-visible detector of dual wavelength was used to analyze biological matrices. For the chromatographic separation, a Hypersil BDS-C18 column (250 mm 4.6 mm, 5 m) (Agilent in Mumbai, India) was employed. The mobile phase was used in isocratic mode at a flow rate of 0.65 mL/min a ratio of 54:46% v/v of ACN and 10 mM KH_2PO_4 buffer (pH 5.0) at. The injection volume of the samples was 20 μL, and the UV detection wavelength was 226 nm. Throughout the whole analysis, the column and auto-sampler temperatures were set to 25 °C.

For both brain matrices and plasma, the bioanalytical methods for RTG were linear between range of 100–1200 ng/g and 100–1200 ng/mL respectively. For plasma, LOD was found to be 9.75 ng/mL. For the brain matrices, LOD was found to be 9.95 ng/mL.
